# Supplementary material for: Low compositions of human toll-like receptor 7/8-stimulating RNA motifs in the MERS-CoV, SARS-CoV and SARS-CoV-2 genomes imply a substantial ability to evade human innate immunity
Source: PeerJ. 2021 Feb 24;9:e11008. doi: 10.7717/peerj.11008 (PMC7912611; doi:10.7717/peerj.11008)
Supplement: Supplemental Information 2 [file peerj-09-11008-s002.docx]

Table S1. Number and accession number of human coronavirus genomic sequences used in this study.

| Virus | Accession Number | Number of Sequences |
| --- | --- | --- |
| 229E | JX503060, MF542265, KF514432, KF514433, KF514430, KU291448, MN306046, KY369913, KY369910, KY369911, KY684760, KY967357, KY983587, KY621348, KY674914, KY369908, KY369909, MN369046, KY369912, KY369914, JX503061, KY674919 | 22 |
| OC43 | KF923918, KF923895, KF923924, KF923925, KF923902, KF923903, KF923904, KP198611, KF923886, KF923889, KF923887, KY014281, KY014282, KP198610, KF923888, KF923893, KF923905, KF923896, KF923898, KF923906, KF923897, KF923899, KF923900, KF923890, KF923891, KF923892, KF923894, KF923907, KF923908, KF923909, KF923910, KF923901, KF923911, KF923912, KF923913, KF923914, KF923915, KF923916, KF923917, KF923919, KF923920, KF923921, KF923922, KF923923, KU131570, MF314143, MH121121, MF374984, MF374985, MF374983, MN306036, MN310478, MN306041, MN306042, MN306043, KY684759, KY967361, KY967360, KY983583, KY967359, KY967358, KY983585, KY967356, KY983588, KY369906, KY369905, MN310476, MN306053, KY369907, JN129835, KJ958218, KJ958219, KX538964, KX538975, KX538976, KX538977, KX538978, KX538979, KX538965, KX538966, KX538967, KX538968, KX538969, KX538970, KX538971, KX538972, KX538973, KX538974, KY554972, KY674917, KY674918, KY554973, KY554974, KY554975, KY674920, KX344031, KF530068, KF530092, KF530060, KF530085, KF530086, KF530077, KF530083, KF530087, KF530073, KF530066, KF530065, KF530061, KF530088, KF530076, KF530096, KF530091, KF530089, KF530067, KF530082, KF530094, KF530095, KF530079, KF530097, KF530074, KF530071, KF530090, KF530084, KF530075, KF530078, KF530063, KF530064, KF530098, KF530099, KF530072, KF530080, KF530069, KF530070, KF530081, MN026164, MG977451, MG977452, FJ415324, JN129834 | 139 |
| HKU1 | KF686343, KF686346, KF430201, AY597011, NC_006577, KF686340, MK167038, KY674943, KY674942, KT779555, KT779556, HM034837, KF686345, KF686341, KY674921, MH940245, KF686339, KF686342,KF430202, KF686344, KF430200, KF430199, KF850450, KF686338, KF430196, KY674941 | 26 |
| NL63 | MG428704, MG428703, MG428702, MG428706, MG428701, MG428705, KT266906, JX504050, KT381875, KU521535, KX179500, KY554971, MK334045, KY554970, JX104161, JX524171, MN306040, MG772808, MK334047, MK334046, KY554967, KY554968, MK334043, MK334044, MG428699, KY829118, KY554969, JQ900259, KY674916, KY983586, JQ765573, JQ765574, JQ765575, JQ765568, JQ765571, JQ765572, JQ765566, JQ765567, JQ900255, MN306018, JQ765569, JQ765570, JQ765564, JQ765565, JQ765563, JQ900260, JQ900257, JQ900256, KF530105, KF530110, KF530114, KF530108, KF530111, KF530109, KF530104, KF530113, KF530107, KF530106, KF530112, JQ900258, KY674915 | 61 |
| MERS | KT029139, JX869059, KU308549, KP209310, KP209308, KP209307, KP209313, KP209309, KP209311, KP209306, KP209312, KF186567, KJ829365, KP223131, KT156560, KT156561, KT026454, KJ813439, KX034094, KX034095, KX034097, KX034098, KX034099, KX034100, KF600651, KF600628, KT026456, KF600647, KF186566, KF192507, KC667074, KT006149, KC164505, KF600632, KT026453, KT026455, KF961221, KF961222, KT806046, KT806044, KT806055, KT806045, KT861628, KT806053, KT806052, KT806048, KU851863, KU851864, KU851862, KU851861, KU851860, KR011266, KR011264, KR011263, KR011265, KT806051, KT806049, KU710264, KT861627, KT806047, KF186564, KF600630, KX034096, KF600652, KF600627, KF600644, KU851859, KF600613, KF600634, KF600645, KF186565, KJ156910, KJ156874, KJ156934, KJ156944, KJ156869, KJ156949, KJ156881, KJ156866, KJ156952, KF600612, KF600620, KM015348, KJ361503, KM210278, KM210277, KC776174, KM027255, KM027256, KM027259, KF745068, KT121577, KT121573, KT121575, KT121572, KT121576, KT121574, KM027262, KJ556336, KJ361501, KM027260, KF958702, KT374052, KT374053, KT374054, KT374055, KT374051, KT374050, KT374056, KT374057, KM027258, KM027261, KT121580, KT121578, KJ361502, KM027257, KT121581, KT121579, KT806054, KU710265, KJ361500 | 121 |
| Other | MH395139, MH432120, MH306207, MH454272, NC_019843, MK052676, MK796425, MN120514, MN120513, MH013216, MK483839, MK462244, MK462247, MK039552, MK039553, MK462248, MK462250, MK462251, KY673148, MK462254, MH029552, MG546330, MG546331, MG366883, MG912601, MK462249, MK462253, MN723544, MK462256, MN365232, MN365233, MG912595, MG912596, MG912597, MG366880, MG912598, MG912599, MG366881, MG366882, MG912600, MG912607, MG912602, MG912604, MG912603, MG912608, MG912605, MG912606, MK462252, KY581684, KY581685, KY581686, KY581687, KY581688, KY581689, KY581690, KY581691, KY581692, KY581693, KY581694, MK129253, MG470650, MK462243, MN723542, MK462245, MK462246, MN723543, NC_038294, MG520076, KY688120, KY688123, KY688124, MG757595, KX154694, KX154690, MG011351, MG011341, MG011352, MG757593, MG757594, MG757596, MG757597, MG757598, MG757599, MG757600, MG757601, MG757602, MG520075, MG757603, MG757605, MG757604, KX154689, MH310911, KX154684, KX154685, KX154686, KX154687, KX154688, KX154691, KX154692, KX154693, MG011340, MG011342, MG011343, MG011347, MG011344, MG011346, MG011345, MG011359, MG011350, MG011349, MG011348, MG011353, MG011354, MG011355, MG011356, MG011358, MG011357, MG366483, MH310910, MG011361, MG011360, MG011362, MH822886, MH310912, KY688122, KY688118, MH310909, KY688119, MK462255, KY688121, MK280984, MF000460, MF000458, MF000457, MF000459, KT326819, KY689142, KT225476 | 138 |
| SARS | MK062183, MK062184, AY390556, AY394995, AY278489, AY313906, DQ640652, AY291451, AY502923, AY502925, AY502926, AY502927, AY502928, AY502929, AY502930, AY502931, AY502932, AY394850, AY278487, AY714217, AY310120, AY291315, AY278491, AY323977, AY502924, AP006557, AP006559, AP006560, AP006561, MK062179, MK062180, MK062181, MK062182, AY274119, AY485278, AP006558, AY485277, AY559096, AY559081, AY461660, AY559085, AY345986, AY345987, AY345988, AY282752, AY278554, AY304495, AY394993, AY394991, AY427439, AY279354, AY278488, AY568539, AY613947, DQ182595, FJ882963, AY559082, AY559086, GU553363, GU553364, AY395003, AY559094, AY395002 | 63 |
| 2019-nCoV | MN988668, MN988669, LC521925, LC522972, LC522973, LC522974, LC522975, MN997409, MN994467, MN994468, MT027062, MT027063, MT027064, MT044258, MT106052, MT106053, MT118835, MT159717, MT159707, MT159708, MT159709, MT159711, MT159712, MT159713, MT159714, MT159715, MT159716, MT184907, MT159718, MT184908, MT184909, MT184910, MT184911, MT184912, MT184913, MT159719, MT159720, MT159721, MT159722, MT159705, MT159706, MT159710, MN988713, MT044257, MT039888, MT106054, MT020880, MT020881, MN985325, MT039887, MN938384, MN975262, MT007544, MT019529, MT019530, MT019531, MT019532, MT019533, MT233526, MT246667, MT039873, MT295464, LC528232, LC528233, LC534418, LC534419, MT126808, MT135041, MT135042, MT135043, MT135044, MT281577, MT253696, MT253697, MT253698, MT253699, MT253701, MT253700, MT253702, MT253703, MT253704, MT253705, MT253706, MT253707, MT253708, MT253709, MT253710, MT123290, MT123291, MT123293, MT123292, MT226610, MT121215, MT093631, MT291831, MT291832, MT291833, MT291834, MT291835, MT291836, MT291826, MT291827, MT291828, MT291829, MT291830, MT259226, MT259231, MT259230, MT259229, MT259228, MT259227, MT049951, MT198652, MT292572, MT292571, MT292569, MT292573, MT292574, MT292575, MT292570, MT292576, MT292577, MT292578, MT292579, MT233519, MT233522, MT233523, MT050493, MT012098, MT276598, MT276597, MT066156, MT072688, MT240479, MT262993, MT263074, MT093571, MT192759, MT276324, MT258377, MT258378, MT258379, MT258380, MT258381, MT258382, MT258383, MT276330, MT276329, MT276326, MT276327, MT276328, MT192765, MT276323, MT276331, MT295465, MT262904, MT262905, MT262906, MT262915, MT262907, MT262908, MT262909, MT262910, MT262896, MT262911, MT262912, MT262913, MT262916, MT262914, MT262897, MT262898, MT262899, MT262900, MT262901, MT262902, MT262903, MT246449, MT246450, MT246451, MT246452, MT246453, MT246454, MT246455, MT246456, MT246457, MT246458, MT246459, MT246460, MT246461, MT246462, MT246464, MT246466, MT246467, MT246468, MT246469, MT246470, MT246471, MT246472, MT246473, MT246474, MT246475, MT246476, MT246477, MT246478, MT246479, MT246480, MT246481, MT246482, MT246484, MT246485, MT246486, MT246487, MT246488, MT246489, MT246490, MT251977, MT251978, MT251972, MT251973, MT251974, MT251975, MT251976, MT251979, MT251980, MT259235, MT259236, MT259237, MT259239, MT259241, MT259243, MT259244, MT259245, MT259246, MT259247, MT259248, MT259249, MT259250, MT259251, MT259252, MT259253, MT259254, MT259256, MT259257, MT259258, MT259260, MT259261, MT259263, MT259264, MT259266, MT259267, MT259268, MT259269, MT259271, MT259273, MT259274, MT259275, MT259277, MT259278, MT259280, MT259281, MT259282, MT259284, MT259285, MT259286, MT259287, MT263381, MT263382, MT263383, MT263384, MT263386, MT263387, MT263388, MT263390, MT263391, MT263392, MT263394, MT263395, MT263396, MT263398, MT263399, MT263400, MT263402, MT263403, MT263404, MT263405, MT263406, MT263408, MT263410, MT263411, MT263412, MT263413, MT263414, MT263415, MT263416, MT263417, MT263418, MT263419, MT263420, MT263421, MT263422, MT263423, MT263424, MT263425, MT263426, MT263428, MT263429, MT263430, MT263431, MT263432, MT263433, MT263434, MT263435, MT263436, MT263437, MT263438, MT263439, MT263440, MT263441, MT263442, MT263443, MT263444, MT263445, MT263446, MT263447, MT263448, MT263449, MT263450, MT263451, MT263452, MT263453, MT263454, MT263455, MT263456, MT263457, MT263458, MT263459, MT263462, MT263463, MT263464, MT263465, MT263467, MT263468, MT263469, MT152824, MT163716, MT163717, MT163718, MT163719, MT276325, MT293176, MT293177, MT293185, MT293188, MT293192, MT293195, MT293174, MT293163, MT293156, MT293158, MT293159, MT293160,MT293161, MT293162, MT293164, MT293165, MT293166, MT293167, MT293168, MT293169, MT293170, MT293171, MT293172, MT293173, MT293175, MT293178, MT293179, MT293180, MT293181, MT293182, MT293183, MT293184, MT293186, MT293187, MT293189, MT293190, MT293191, MT293194, MT293196, MT293197, MT293198, MT293199, MT293200, MT293201, MT293202, MT293204, MT293205, MT293206, MT293207, MT293208, MT293209, MT293210, MT293211, MT293212, MT293213, MT293214, MT293215, MT293216, MT293218, MT293219, MT293220, MT293222, MT293224, MT293225, MT192772, MT192773, MT066175, MT066176, MT039890, LC529905, LR757995, LR757996, LR757997, LR757998, MT188341, MT188340, MT188339, MN996527, MN996528, MN996529, MN996530, MN996531, MN908947 | 432 |
|  | Total | 1002 |
