# Supplementary material for: Low compositions of human toll-like receptor 7/8-stimulating RNA motifs in the MERS-CoV, SARS-CoV and SARS-CoV-2 genomes imply a substantial ability to evade human innate immunity
Source: PeerJ. 2021 Feb 24;9:e11008. doi: 10.7717/peerj.11008 (PMC7912611; doi:10.7717/peerj.11008)
Supplement: Supplemental Information 3 [file peerj-09-11008-s003.docx]

Table S2. Number and accession number of coronavirus genomic sequences from non-human hosts used in this study.

| Virus | Accession Number | Number of Sequences |
| --- | --- | --- |
| Bat | MH938448, MH938450, MH938449, MH687934, MH687935, MH687936, MH687937, MH687938, MH687939, MH687940, MH687941, MH687942, MH687943, MH687944, MH687945, MH687946, MH687947, MH687948, MH687949, MH687950, MH687951, MH687952, MH687953, MH687954, MH687955, MH687956, MH687957, MH687958, MH687959, MH687960, MH687961, MH687962, MH687963, MH687964, MH687965, MH687966, MH687967, MN065811, MG923574, NC_022103, KY799179, KJ473806, NC_028811, KJ473809, NC_028833, KJ473810, KJ473807, NC_028814, KJ473808, NC_028824, MK211374, MK211373, MK211375, MK211376, MK211377, MK211378, MK211379, MK211369, MK211370, MK211371, MK211372, MN611525, MN611523, KX442565, KX442564, KJ473821, MG596802, MG596803, MF593268, MN611518, MN611524, KY073746, KY073744, NC_032107, KY073745, MN611520, MN611522, MK720945, MK720946, MN611517, KU762338, NC_030886, KT444582, JX993988, JX993987, KJ473812, KJ473811, KJ473813, KJ473815, KJ473814, KJ473816, MN611521, MF094687, MF094688, MF094685, MF094686, MK720944, MN611519, KJ473795, KJ473796, KY770858, KY770859, KY770850, KY770851, MK492263, MG693170, MG693168, MG693172, MG693169, KY770860, KU182965, KU182964, KX574227, NC_034440, LC469308, GU190215, NC_014470, KF430219, JQ989269, JQ989267, JQ989268, JQ989272, JQ989273, JQ989266, JQ989271, JQ989270, NC_018871, KF636752, NC_025217, KT253272, KT253270, KT253269, KT253271, KY073747, KY073748, KC869678, KJ473799, KJ473797, KJ473800, KJ473798, EF065510, EF065511, EF065512, EF065509, KJ473820, NC_009020, EF065514, EF065515, EF065516, EF065513, HM211100, HM211101, HM211098, HM211099, NC_009021, MG762674, DQ022305, DQ412042, DQ412043, DQ071615, MN996532, KY417142, MG772933, MG772934, KF294457, KY417145, KY417143, KY417144, KY417146, KY417147, KY417148, KY417149, KY417150, KY417151, KY417152, KC881006, KC881005, KF367457, KP886808, KP886809, KF569996, FJ588686, Y352407, KU973692, EF065506, EF065507, EF065508, EF065505, KJ473822, NC_009019 | 190 |
| Camel | KT368902, KT368903, KT368904, KT368905, KT368906, KT368909, KT368912, KT368892, KT368893, KT368910, KT368894, KT368911, KT368898, KT368899, KT368900, KT368901, KT368897, KT368895, KT368896, KT368907, KT368908, KT368915, KT368916, KT368913, KT368914, MF593476, KT368891, MK357908, MK357909, MK564474, MK564475, KY673149, KY581695, KY581696, KY581697, KY581698, KY581699, KY581700, MF598702, MF598713, MF598700, MF598703, MF598711, MF598699, MF598709, MF598705, MF598716, MF598708, MF598712, MF598701, MF598704, MF598714, MF598706, MF598719, MF598707, MF598722, MF598715, MF598720, MF598721, MF598710, MF598717, MF598687, MF598688, MF598689, MF598690, MF598691, MF598692, MF598693, MF598694, MF598695, MF598696, MF598602, MF598697, MF598698, MF598603, MF598604, MF598605, MF598606, MF598607, MF598608, MF598609, MF598610, MF598611, MF598594, MF598612, MF598613, MF598614, MF598615, MF598616, MF598617, MF598618, MF598619, MF598620, MF598621, MF598595, MF598622, MF598623, MF598624,MF598625, MF598626, MF598627, MF598629, MF598630, MF598631, MF598632, MF598633, MF598634, MF598635, MF598636, MF598637, MF598638, MF598639, MF598640, MF598596, MF598641, MF598643, MF598644, MF598645, MF598646, MF598647, MF598648, MF598649, MF598597, MF598650, MF598651, MF598652, MF598653, MF598654, MF598655, MF598656, MF598657, MF598658, MF598659, MF598598, MF598660, MF598661, MF598662, MF598663, MF598664, MF598665, MF598666, MF598667, MF598668, MF598669, MF598599, MF598670, MF598671, MF598672, MF598673, MF598674, MF598675, MF598676, MF598677, MF598678, MF598600, MF598679, MF598680, MF598681, MF598682, MF598683, MF598684, MF598685, MF598686, MF598601, MH259485, MH259486, MN507638, MG923470, MG923473, MG923471, MG923466, MG923467, MG923468, MH734114, MH734115, MG923469, MG923472, MG923474, MG923475, MG923478, MG923479, MG923480, MG923476, MG923481, MG923477, MK967708, KJ477103, MN514962, MN514963, MN514964, MN514965, MN514966, MN514967, KF906249, KF906250, KF906251, JQ410000, MF593473, NC_028752, KT253327, KT253325, KT253324, KT253328, KT253326, KU291449, KT368827, KT368828, KT368829, KT368830, KT368831, KT368832, KT368833, KT368834, KT368835, KT368836, KT368837, KT368838, KT368839, KT368840, KT368841, KT368842, KT368843, KT368844, KT368845, KT368824, KT368858, KT368866, KT368867, KT368859, KT368860, KT368861, KT368862, KT368863, KT368864, KT368865, KT368846, KT368847, KT368848, KT368849, KT368850, KT368851, KT368852, KT368853, KT368854, KT368855, KT368856, KT368857, KJ650098, KT368868, KT368869, KT368870, KT368871, KT368872, KT368873, KT368874, KT368875, KT368825, KT368876, KT368877, KT368878, KT368826, KT368879, KT368889, KT368890, KT368882, KT368883, KT368880, KT368884, KT368881, KT368885, KT368886, KT368887, KT368888, KP719928, KP719929, KP719930, KP719927, KP719933, KP719932, KP719931, KU242424, KU242423, KX108944, KX108937, KX108946, KX108945, KX108939, KX108938, KT751244, KX108940, KX108941, KX108942, KX108943, KJ650297, KJ650295, KJ650296, KJ713298, KJ713299, KJ713296, KJ713297, KJ713295, KT877351, KT877350, KF917527, KJ477102 | 305 |
| Cat | MH817484, KP143512, KP143507, KP143508, KP143509, KP143510, KP143511, KX722530, KU215424, KU215425, KU215420, KU215421, KU215422, KU215426,KU215427, KU215428, KU215423, JQ408980, KF530271, KF530270, KF530122, KF530119, KF530121, KF530133, KF530129, KF530135, KF530132, KF530116, KF530136, KF530134, KF530125, KF530115, KF530130, KF530126, KF530131, KF530120, KF530127, KF530124, KF530118, KF530117, KF530123, MG893511, DQ010921, KY292377, KY566209, KY566210, KY566211, KU215419, KX722529, MN165107, HQ392470, HQ392471, HQ392472, HQ012372, HQ392469, JN183882, JN183883, JQ408981, KC461235, KC461237, KC461236, GQ152141, FJ938051, FJ938059, FJ938052, FJ938057, FJ938058, HQ012367, HQ012368, FJ938060, HQ012369, GU553361, GU553362, HQ012370, FJ938061, HQ012371, FJ938054, FJ938056, FJ938053, FJ938055, FJ938062 | 81 |
| Civet | AY572034, AY304488, AY304486, AY545918, AY545917, AY545916, AY545914, AY545915, AY686863, AY686864, AY572035, AY572038, AY515512, AY613948, AY613949, AY613950, FJ959407 | 17 |
| Dog | JQ404409, KC175339, JN856008, KP981644, GQ477367, KY063616, KY063617, KY063618, KC175340, KC175341, JQ404410, KX432213, JX860640 | 13 |
| Pig | LC216914, LC216915, NC_039208, LC260038, MN025260, KY513724, KY513725, KY293677, KY293678, MF280390, KY363867, KY363868, MG242062, MF095123, MG832584, MN249445, MK355396, MF642322, MF642323, MF642324, MF642325, MK211169, MK330604, MK330605, MK993519, KY354363, KY354364, MF431742, LC260039, LC260040, LC260045, MH708123, MH708124, MH708125, LC260041, KY364365, MG837131, MG837130, KY926512, LC260042, LC260043, KX998969, MH715491, MK572803, MF431743, MF041982, MK005882, LC260044, MF769440, MF769423, MF769424, MF769430, MF769439, MF769425, MF769419, MF769431, MF769422, MF769418, MF769421, MF769420, MF769436, MF769429, MF769417, MF769435, MF769434, MF769433, MF769438, MF769428, MF769437, MF769416, MF769427, MF769441, MF769426, MF769432, MF769442, MF769443, MF769444, MF094681, MF094682, MF094683, MF094684, MK651076, MH615810, MG605090, MG605091, MG557844, MK977618, KR270796, DQ811787, NC_028806, DQ811785, DQ811788, HM776941, KX058075, FJ755618, KX083668, KT696544, NC_038861, KP202848, KX499468, KC962433, KU729220, DQ201447, HQ462571, DQ811786, DQ811789, AJ271965, MF083115, KY994645, KY419104, KY419105, KY419107, KY419110, KY419113, KY419109, KY419111, KY419112, KY419106, KY419103, KJ481931, KJ601777, KJ601778, KJ601779, KJ601780, JQ065043, JQ065042, NC_016990, KJ584355, KJ569769, KJ584357, KJ620016, KM012168, KJ584359, KT381613, KJ462462, KJ584358, KJ584356, KT266822, KT021234, KP757890, KP757891, KT336560, KP757892,KY065120, KU981059, KX118627, KR131621, KU051641, KU051649, KX834352, KX834351, KX022602, KX022603, KX022604, KX022605, KU984334, KR150443, KR265856, KR265857, KR265852, KR265851, KR265865, KR265849, KR265850, KR265853, KR265859, KR265848, KR265864, KR265847, KR265854, KR265855, KR265860, KR265861, KR265858, KR265862, KR265863, KJ567050, KM820765, KJ769231, KF267450, KR003452, KX839246, MN315264, KC210145, KU646831, MN486588, JX188454, MK584552, LC063810, LC063837, LC063833, MK559454, MK559456, MK559455, JQ023162, MF807952, JN825712, KX066126, MF807951, KR265831, MK606368, MK606369, KY649107, KU380331, JQ282909, KC140102, KR153325, KR153326, KX016034, KY793536, MH581489, KR809885, KT199103, KR095279, KP890336, KX981440, MF346935, KF760557, KJ526096, KX058031, KX058033, KX058032, MF375374, JN547228, MH061339, KU975389, MH061336, MH061341, MH061343, MH061337, MH061342, MH061340, MH061338, MF462814, MK140811, MK140812, MK140813, MK140814, KF840537, KC196276, KY928065, JX261936, KM887144, MG837011, MG837012, KX791060, KJ020932, MK796238, KU558702, MK071625, MK071619, MK071620, MK071629, MK071630, MK071631, MK071633, MK071635, MK071636, MK071637, MK071638, MK071639, KU569509, MK071626, MK071621, MK071623, MK071624, MK071622, MK071632, MK071627, MK071628, MK071634, LC063812, MH708895, LC063811, KP765609, KR011756, MN056942, MK138353, MK644601, JX647847, JX112709, JX088695, KM089829, MH726362, MH726384, MH726370, MH726385, MH726408, MH107321, MH726374, MH726387, MH726373, MH726393, MH726391, MH726386, MH726364, MH726376, MH726375, MH726363, MH726371, MH726368, MH107322, MH726366, MH726365, MH726367, MH726380, MH726372, MH726377, MH726369, MH726394, MH726395, MH726379, MH726378, MH726396, MH726388, MH726398, MH726397, MH726389, MH726390, MH726401, MH726399, MH726381, MH726392, MH726400, MH726402, MH726382, MH726405, MH726406, MH726383, MH726403, MH726407, MH726404, LC063823, LC063829, MH708243, KP688354, MK862249, KP403802, MK690502, LC063825, KT941120, KX289955, KF468753, KF468754, LC063820, LC063821, LC063841, LC063842, LC063819, LC063831, LC063832, LC063843, KF650370, KF650371, KF650372, KF650373, KF650374, KF650375, KR061458, LC063834, LC063839, LC063835, LC063813, KX534206, MH910099, KC210147, KC210146, KY070587, KR818833, KX534205, MH056658, KJ623926, KX580953, LC063844, LC063845, LC063814, LC063815, LC063826, LC063824,LC063817, LC063818, KJ662670, KM403155, KR873431, KR873435, KR873434, KY825240, KY825241, KY825242, KY825243, KY963963, MH052681, MH052682, MH052683, MH052684, MH052685, MH052687, MH052688, MH052689, MH243316, MH243318, MH243319, MK032689, MK032690, MK032691, MK032692, MK482396, MK482397, KJ588064, KJ588063, KJ588062, MF737355, MK644602, JX489155, MK392335, KJ777677, MK644603, KJ645708, KJ645700, KR265766, LC063827, KF468752, LC063838, LC063846, MK644604, MF281416, LC063830, LC063840, KJ778615, KJ778616, KM052365, MF782686, MF782687, KP641661, KJ408801, KJ584361, KJ399978, KP641662, KP641663, LC063836, LC063816, LC063847,KM189367, KR078300, KR078299, KY499262,KU893870, KU893871, KU893872, KU893873, KU893861, KU893869, MG837058, KY111278, MH748550, MK841494, MK841495, KM609206, KM609207, KM609208, KM609203, KM609204, KM609205, KM609209, KY007139, KY007140, KM609211, KM609210, KM609212, KY420075, KM609213, MH006959, MH004412, MH006961, MH004416, MH006964, MH004417, MH004413, MH004418, MH004419, MH004420, MH004415, MH004414, MH006957, MH006965, MH006962, MH006958, MH004421, MH006963, MH013465, MH013466, MH013462, MH006960, MH013463, MH013464, KU982969, KU982967, KU982974, KU982981, KU982980, KU982972, KU982979, KU982973, KU982971, KU982975, KU982978, KU982970, KU982968, KU982976, KU982977, KU982966, MG781192, KY929405, KY929406, MH891590, MH891587, MH891584, MH891585, MH593900, MH891589, MH891586, KP162057, JX560761, KX064280, MH117940, MK558089, KJ196348, KU297956, KY019623, KY019624, MK702008, KP728470, MK644605, KM392232, KM392226, KM392227, KM392229, KM392230, KM392231, KM392224, KM392225, KM392228, KY499261, LC022792, LC063822, LC063828, MK673545, KP403954, KC189944, KJ960178, KJ960179, KJ960180, KR610991, KR610992, KR610993, KR610994, KT591944, KF272920, KR265823, KJ645638, KR265812, KJ645651, KR265761, KM975738, KM975739, KM975736, KM975740, KT860508, KR265763, KR265815, KR265769, KR265795, KR265770, KR265817, KR265826, KR265785, KR265802, KR265786, KR265796, KR265789, KR265782, KR265804, KJ645659, KJ645675, KJ645680, KJ645689, KJ645690, KF452323, KJ645635, KR265800, KR265801, KJ645641, KF452322, KF804028, KJ645694, KJ645695, KJ645696, KR265805, KR265808, KJ645649, KJ645636, KR265827, KJ645666, KJ645688, KJ645701, KR265814, KR265777, KR265797, KJ645637, KJ645643, KR265819, KR265767, KJ645650, KR265772, KR265791, KJ184549, KR265807, KR265822, KJ645691, KJ645703, KR265799, KR265764, KR265768, KR265759, KR265825, KR265816, KR265760, KR265793, KR265794, KR265776, KR265828, KR265783, KR265813, KR265778, KR265773, KR265798, KR265818, KR265788, KR265829, KR265806, KR265775, KR265843, KR265832, KR265771, KR265841, KR265842, KJ645647, KJ645648, KJ645704, KJ645652, KJ645655, KJ645656, KJ645705, KJ645658, KJ645660, KJ645661, KJ645663, KJ645706, KJ645667, KJ645668, KJ645671, KJ645672, KJ645673, KJ645674, KJ645676, KJ645677, KJ645707, KJ645678, KJ645679, KJ645681, KJ645682, KJ645686, KJ645687, KJ645692, KJ645693, KR265821, KR265779, KR265846, KR265820, KR265844, KJ645684, KJ645685, KM975741, KM975735, KM975737, KR265809, KR265765, KR265803, KR265784, KJ645646, KJ645662, KJ645683, KJ645698, KJ645699, KJ645702, KR265840, KR265781, KR265830, KR265780, KJ645657, KJ645664, KJ645665, KJ645670, MG334554, MG334555, KR265762, KJ645640, KR265810, KJ645642, KJ645644, KR265792, KR265845, KR265824, KR265833, KR265787, KR265811, KR265774, KJ645654, KJ645697, KJ645639, KJ645645, KR265790, KR265834, KJ645653, KJ645669, MK138516, JQ023161, MN037494, KX812523, KX812524, KR818832, MH891588, KU252649, KT021227, KT021232, KT021228, KT021233, KT021229, KT021230, KT021231, MK250953, KX550281, MK409659, MK409657, MK409658, JX524137, KU664503, KU558701, KU847996, MH539766, MH697599, MF167434, MF370205, AY654624, KR061459 | 755 |
| Avian | MG812377, MG812375, MG812376, MG812378, AX154950, KY626044, MH021175, MK071267, KY933090, KY626045, MK204393, MK204411, FJ376620, KR822424, LN610099, KF377577, MK142676, MH779856, MH779857, MH779858, MH779859, MH779860, NC_001451, KF696629, LP994923, LQ302036, LY505956, LY611929, MA765825, FN430414, FN430415, NC_010800, AJ311317, AY646283, EU418975, EU418976, DQ001338, DQ001339, M95169, AY692454, AY319651, AY514485, EU637854, FJ807652, AY338732, DQ834384, AY851295, AY641576, DQ288927, DQ646405, EU095850, EU022525, EU022526, GQ427175, GQ427176, GQ427174, GQ427173, JQ065045, NC_016992 | 59 |
| Chicken | KY933089, KX258195, MG913343, MG913342, KX266757, KX272465, GQ504720, GQ504721, KR231009, KT203557, MF924724, FJ904715, MK329221, KU361187, KU361188, MN197549, KT946798, MK309398, MG197727, MF882923, MH020185, KF663559, KF663560, KF663561, KX348117, KX348115, KX389094, KP118882, KP118881, KX364295, KX348116, KX364294, KX364298, KX348114, JX195175, JF828981, KJ425485, KJ425486, KX236008, KP790143, KX236015, KT736031, KX077987, JX195177, JX195178, KP118894, KC013541, KC119407, KX219792, KX185058, KX302874, KF411041, KX640829, KP343691, KX236016, JF330898, KX400753, KJ425487, MK574042, MK574043, KJ425488, KX219793, KJ425489, KX247127, KJ425490, KJ425491, KX247130, KJ425492, KX236014, KJ425493, KP036503, KJ425494, KJ425495, KP118883, MG448607, KX247129, KX247128, KJ425496, KP118889, KP118890, KX252774, KJ425497, KX252775, KP036504, KJ425498, KP118880, KJ425499, KJ425500, KJ425501, KJ425502, KX252776, KP118887, KX252773, KX252781, KX219791, JF274479, KX252788, KX252783, KX252790, KX252789, KX302869, KJ425503, KJ425504, JF828980, KJ425505, KX375806, KX375807, KX275392, KP868572, KJ425506, KP118891, KX372249, KX434788, KX275393, KX302868, KX302870, KJ425507, KP790144, KP790146, KP036502, KX275391, KX275390, KP790145, KX236004, KX185057, KX375808, KJ425508, KX259248, KX259253, KX302871, KX302866, KX252778, KX364293, KX236005, KX364296, KX259249, KX302875, KX364290, KC136209, KC506155, KJ425509, MK937832, KP868573, KP036505, KX364297, KX434789, KX425847, MK937833, KX302873, KX302867, KX375805, KX219794, KX302862, KX259250, KX259251, KX259252, KX259255, KX434790, KX259256, KX259257, KX302861, KX302865, KX252779, KX252782, KX252780, KX252784, KF411040, KX252786, KP118892, KP118888, KX252787, KX252791, JF330899, KY799582, KX236001, KX252777, KX259254, KX302863, KX302864, KX236012, KX236011, KX236006, KX252785, KX236007, KX236010, KX302872, KX364299, KX364291, KP118893, KJ425510, KJ425511, KJ425512, KX364292, KP118884, KP118885, KX236000, KX236003, KX236002, KJ435286, KX364300, KJ435283, KP118886, KJ435284, KX219801, KX236013, KX219798, KJ435285, KX372250, KX219799, KX219800, KX219795, KX302860, KX275394, KX252772, KX236009, KX219797, KX219796, JX195176, KU356856, KJ128295, KF668605, KM213963, MN096598, JQ088078, KP662631, KT736032, FJ904718, FJ904719, HM245924, MK878536, HM245923, MK644086, MN599049, MG021194, KY776701, KY407557, KY407556, KY407558, MK032181, MH924835, KY776700, MK032178, MK032179, MK032177, MK032180, KY620116, KP780179, MK581203, MK581206, MK581204, MK581205, KT886454, MK581200, MK581201, MK581202, KY047602, MK581207, MK581208, GQ504722, GQ504723, KC008600, JX897900, HQ848267, HQ850618, MG517474, MK937831, EU817497, MH181793, MK217372, MK937829, MK217373, MK217374, MK937828, MK217375, MG738154, MG738155, MG763935, MN512434, MN512435, MN512436, MN512437, MN512438, KY805845, KY805846, MH539771, MH539772, KJ135013, KR902510, MG233398, MF924725, JQ977698, KX185056, KR608272, KX185059, MK937830, MK728875, FJ904720, FJ904721, FJ904722, FJ904723, FJ904713, GQ504725, GQ504724, KM586818, KU900739, KU900740, KU900744, KU317090, JF732903, KY421673, KF574761, KF853202, KU900743, KU900742, KU900738, KU900741, JQ977697, KY421672, KT852992, GU393331, GU393332, GU393333, GU393334, GU393335, GU393336, GU393337, MF421319, MF421320, MH878976, KF931628, KF460437, JF893452, JX840411, MF508703, EU714028, EU526388, FJ904714, FJ904717, FJ904716, FJ888351, EU714029, GU393338 | 342 |
|  | Total | 1762 |
