# Supplementary material for: Low compositions of human toll-like receptor 7/8-stimulating RNA motifs in the MERS-CoV, SARS-CoV and SARS-CoV-2 genomes imply a substantial ability to evade human innate immunity
Source: PeerJ. 2021 Feb 24;9:e11008. doi: 10.7717/peerj.11008 (PMC7912611; doi:10.7717/peerj.11008)
Supplement: Supplemental Information 4 [file peerj-09-11008-s004.docx]

Table S3. Sequences of human TLR 7/8 stimulatory ORNs from research reports.

| Name | Sequence | Cell | Cytokine | PubMed ID |
| --- | --- | --- | --- | --- |
| 2.2 sense | GCAUGCGACCUCUGUUUGA | pDC | IFN-α | 19454678 |
| 9.1 sense | UGGACGGCAACUGUUAUUA | Monocyte | IL-12p70 | 19454678 |
| 9.1 antisense | UAAUAACAGUUGCCGUCCA | Monocyte | IL-12p70 | 19454678 |
| 9.2 sense | AGCUUAACCUGUCCUUCAA | Monocyte | IL-12p70 | 19454678 |
| 9.2 antisense | UUGAAGGACAGGUUAAGCU | Monocyte | IL-12p70 | 19454678 |
| 9.3 sense | ACCUGUCCUUCAAUUACCA | Monocyte | IL-12p70 | 19454678 |
| 9.3 antisense | UGGUAAUUGAAGGACAGGU | Monocyte | IL-12p70 | 19454678 |
| Ass | AGCUUAACCUGU | Monocyte | IFN-α | 19454678 |
| Bss | AACCUGUCCUUC | Monocyte | IFN-α | 19454678 |
| Lss | AGCUUAACCUGUCCUU | Monocyte | IFN-α | 19454678 |
| Rss | UUAACCUGUCCUUCAA | Monocyte | IFN-α | 19454678 |
| P20 | UUGAAGGACAUGUCCUUCAA | PBMC | IFN-α | 19454678 |
| P20-3M | UGUCAGGACAUGUCCUUCAA | PBMC | IL-12p70 | 19454678 |
| P20-5M | UGUCCUGACAUGUCCUUCAA | PBMC | IL-12p70 | 19454678 |
| P20-6M | UGUCCUUACAUGUCCUUCAA | PBMC | IL-12p70 | 19454678 |
| P20-20M | UGUCCUUCAAUGUCCUUCAA | PBMC | IL-12p70 | 19454678 |
| 27+2 sense | GCUGACCCUGAAGUUCAUCUGCACCACUU | pDC | IFN-α | 19454678 |
| 27+2 antisense | GUGGUGCAGAUGAACUUCAGGGUCAGCUU | pDC | IFN-α | 19454678 |
| 27+0 sense | AAGCUGACCCUGAAGUUCAUCUGCACC | pDC | IFN-α | 19454678 |
| 27+0 antisense | GGUGCAGAUGAACUUCAGGGUCAGCUU | pDC | IFN-α | 19454678 |
| RNA40 | GCCCGUCUGUUGUGUGACUC | PBMC | TNFα, IL-12p40, IFN-α | 14976262 |
| RNA42 | ACCCAUCUAUUAUAUAACUC | PBMC | TNFa, IL-12p40 | 14976262 |
| ORNs 9.2dr | UGUCCUUCAAUGUCCUUCAA | pDC | Th1 cytokines | 17132722 |
| ORNs 9.2as | UUGAAGGACAGGUUAAGCU | pDC | Th1 cytokines | 17132722 |
| ORNs TLR4.1s | UACUUAGACUACUACCUCG | pDC | Th1 cytokines | 17132722 |
| RNA9.3as | UGGUAAUUGAAGGACAGGU | NK Cell | INF-γ | 19890064 |
| Influenza virus | A AUAAUUGACCUGCUUUCGCU | PBMC | TNFa, IL-12p40, IFNa | 18072859 |
| RSV | UUGUACGCAUUUUUUCGCGU | PBMC | TNFa | 18072859 |
| Measles virus | CUUACCCAACUUUGUUUGGU | PBMC | TNFa | 18072859 |
| Sendai virus | UGUUUUUUCUCUUGUUUGGU | PBMC | TNFa, IFNa | 18072859 |
| Rabies virus | UUGAUCUGGUUGUUAAGCGU | PBMC | TNFa | 18072859 |
| VSV | AAUGGUUUGUUUGUCUUCGU | PBMC | TNFa | 18072859 |
| 27S | GUCCGGGCAGGUCUACUUU | Monocyte | TNFa | 17725606 |
| B-406-AS | UAAUUCGCGUCUGGCCUUCUU | PBMC | TNFa, IFNa | 18250417 |
| B-406-S | GAAGGCCAGACGCGAAUUAUU | PBMC | TNFa, IFNa | 18250417 |
| Lamin-AS | UGUUCUUCUGGAAGUCCAG | PBMC | TNFa, IFNa | 18250417 |
| si9.2-S | AGCUUAACCUGUCCUUCAA | PBMC | IFNa | 18250417 |
| STAT-2AS | GUUCCAUUGGCUCUGGUGCUU | PBMC | TNFa, IFNa | 18250417 |
| GFP21-AS | GAUGAACUUCAGGGUCAGCUU | PBMC | IFNa | 18250417 |
| SA | GAAGGCCUUACGCGAAUUAUU | PBMC | TNFa | 18250417 |
| SB | GAAGGCCUUACGCGAACAAUU | PBMC | TNFa | 18250417 |
| U24 | CUACGAUCGGUCGACCUCGAGUGUGGUGCAGAUGGAUGUAAAAGAAUAUUUGCUAUCUGAGAGAUGGUGAUGACAUUUUAAACCACCAAGAUCGCUGAUGCACCAG | B-cell | INF | 23019335 |
| A-block | CGAGCUGGUGCAGAUGGAUGUAAAAGAAUAAUUUGCUAAUCUGAAGAGAAUGAGUGAUGACAUUUUAAACCACCAAGAUCGCUGAUGCACCAG | B-cell | INF | 23019335 |
| Inv | CGAGCUGGUGCAGAUGGAUGUAAAAGUAAAUUUGUCAUCUGAAGAGUGGUGAUGACAUUUUAAACCACCAAGAUCCGUGAUGCACCAG | B-cell | INF | 23019335 |
| U11snRNA | AUAAUUUUUUGGUAUUU | Splenocytes | autoimmune | 31694883 |
| Sa19 | GGACGGAAAGACCCCGUGG | THP-1 | TNF | 26545385 |
| mtPTL | AGACGAGAAGACCCUAUGG | THP-1 | TNF | 26545385 |
| HsmtD1 | CGCAAGGGAAAGAUGAAAA | THP-1 | TNF | 26545385 |
| HsmtD3_4 | UCAUAAGGAAAGGUUAAAA | THP-1 | TNF | 26545385 |
| BtmtD3_4 | AUCUAAGGAAAGAUUAAAA | THP-1 | TNF | 26545385 |
| ssN | GCGCCGAGGUGAAGUUCGAGGGCGA | PBMC | TNFα, IFNα | 24227841 |
| ssU | GCGCCGAGUUUUAGUUCGAGGGCGA | PBMC | TNFα, IFNα | 24227841 |
| 1170 | GGACUGCGUUCGCGCUUUCC | PBMC | TNFa, IFNa | 16330816 |
| 1171 | GGCUUAUCCAUUGCACUCCGGA | PBMC | TNFa, IFNa | 16330816 |
| 1172 | GACUAGCUUGCUGUUU | PBMC | TNFa, IFNa | 16330816 |
| 1174 | UUUGUGGUAGUGGGGGACUG | PBMC | TNFa, IFNa | 16330816 |
| 1176 | ACGAAGGUGGUUUUCCCAG | PBMC | TNFa, IFNa | 16330816 |
| 1274 | GGACUGCGUUGUGGCUUUCC | PBMC | TNFa, IFNa | 16330816 |
| 1300 | GAUACUUACCUG | PBMC | TNFa, IFNa | 16330816 |
| 1330 | AAUUUUUGA | PBMC | TNFa, IFNa | 16330816 |
| 1332 | AAUUUGUGG | PBMC | TNFa, IFNa | 16330816 |
| 1337 | GUAGUGUUUGUGGGGGACUG | PBMC | TNFa, IFNa | 16330816 |
| 1338 | GUAGUGGGGGACUGUUUGUG | PBMC | TNFa, IFNa | 16330816 |
| 1563 | GACUAGCCUUU | PBMC | TNFa, IFNa | 16330816 |
| 1-as | AAUUUUGAGAAGAUGAUC | PBMC | TNFa | 16609928 |
| 2-s | GACUUGAGCGAGCGCUUUU | PBMC | TNFa | 16609928 |
| 3-s | GUCCGGGCAGGUCUACUUU | PBMC | TNFa, IFNa | 16609928 |
| 4-s | CCAUCGGAUUGUUCUUUCU | PBMC | TNFa | 16609928 |
| 5-as | UGCUAUUGGUGAUUGCCUC | PBMC | TNFa, IFNa | 16609928 |
| R-1075 | CCGUCUGUUGUGUGACUC | PBMC | TNFa, IFNa | 18322178 |
| R-0001 | UAUAUAUAUAUAUAUAUAUA | PBMC | TNFa | 18322178 |
| R-0002 | UUAUUAUUAUUAUUAUUAUU | PBMC | TNFa | 18322178 |
| R-0003 | UUUAUUUAUUUAUUUAUUUA | PBMC | TNFa, IFNa | 18322178 |
| R-0004 | UUUUAUUUUAUUUUAUUUUA | PBMC | TNFa, IFNa | 18322178 |
| R-0005 | UGUGUGUGUGUGUGUGUGUG | PBMC | TNFa, IFNa | 18322178 |
| R-0006 | UUGUUGUUGUUGUUGUUGUU | PBMC | TNFa, IFNa | 18322178 |
| R-0007 | UUUGUUUGUUUGUUUGUUUG | PBMC | TNFa, IFNa | 18322178 |
| R-0008 | UUUUGUUUUGUUUUGUUUUG | PBMC | TNFa, IFNa | 18322178 |
| R-1312 | GCCACCGAGCGUUGUGUACC | PBMC | TNFa, IFNa | 18322178 |
| R-1321 | GCCACCGAGCAUUGUGAACC | PBMC | TNFa, IFNa | 18322178 |
| R-1322 | GCCACCGAGCAUUGAGAACC | PBMC | TNFa, IFNa | 18322178 |
| R-1323 | GCCACCGAGCAAUGAGAACC | PBMC | TNFa | 18322178 |
| R-1324 | GCCACCGAGCAAGGUGAACC | PBMC | TNFa | 18322178 |
| ORN R-1935 | UUGUUGUUGUUGUUGUGGGGG | PBMC | INF-α, INF-γ | 22196370 |
| ORN R-2176 | UUGUUGUUGUUGUUGU | PBMC | INF-α, INF-γ | 22196370 |
| ORN R-2336 | GACACACACACUCACACACACACA | PBMC | INF-α, INF-γ | 22196370 |
| Poly-U | UUUUUU | pDC | IFN-α | 17111347 |
|  | UUGU | PBMC | TNFa, IFNa | 18322178 |
|  | UUUC | PBMC | TNFa, IFNa | 18322178 |
|  | UGUU | PBMC | TNFa, IFNa | 18322178 |
|  | CUGU | PBMC | TNFa, IFNa | 18322178 |
|  | UAUU | PBMC | TNFa, IFNa | 18322178 |
|  | UUUT | PBMC | TNFa, IFNa | 18322178 |
|  | TUGU | PBMC | TNFa, IFNa | 18322178 |
|  | GUUU | PBMC | TNFa, IFNa | 18322178 |
|  | AUUU | PBMC | TNFa, IFNa | 18322178 |
|  | AUGU | PBMC | TNFa, IFNa | 18322178 |
|  | CUUU | PBMC | TNFa, IFNa | 18322178 |
|  | UUUA | PBMC | TNFa, IFNa | 18322178 |
|  | UUGC | PBMC | TNFa, IFNa | 18322178 |
|  | UCUU | PBMC | TNFa, IFNa | 18322178 |
|  | TUUC | PBMC | TNFa, IFNa | 18322178 |
|  | AUUC | PBMC | TNFa, IFNa | 18322178 |
|  | UUCT | PBMC | TNFa, IFNa | 18322178 |
|  | UCUC | PBMC | TNFa, IFNa | 18322178 |
|  | TUUU | PBMC | TNFa, IFNa | 18322178 |
|  | UUGA | PBMC | TNFa, IFNa | 18322178 |
|  | UAUA | PBMC | TNFa, IFNa | 18322178 |
|  | GUUC | PBMC | TNFa, IFNa | 18322178 |
|  | AUAU | PBMC | TNFa, IFNa | 18322178 |
|  | AUAC | PBMC | TNFa, IFNa | 18322178 |
|  | UAUC | PBMC | TNFa, IFNa | 18322178 |
|  | GCUC | PBMC | TNFa, IFNa | 18322178 |
|  | CUAC | PBMC | TNFa, IFNa | 18322178 |
|  | GUAC | PBMC | TNFa, IFNa | 18322178 |
|  | GUGC | PBMC | TNFa, IFNa | 18322178 |
|  | CUGC | PBMC | TNFa, IFNa | 18322178 |
|  | UUCU | PBMC | TNFa, IFNa | 18322178 |
|  | AUAA | PBMC | TNFa, IFNa | 18322178 |
|  | CUAA | PBMC | TNFa, IFNa | 18322178 |
|  | UUUU | PBMC | TNFa, IFNa | 18322178 |
|  | AUUA | PBMC | TNFa, IFNa | 18322178 |
|  | UUAU | PBMC | TNFa, IFNa | 18322178 |
|  | CUCC | PBMC | TNFa, IFNa | 18322178 |
|  | AUCC | PBMC | TNFa, IFNa | 18322178 |
|  | GUCC | PBMC | TNFa, IFNa | 18322178 |

**References:**

1. Ablasser A, Poeck H, Anz D, Berger M, Schlee M, Kim S, Bourquin C, Goutagny N, Jiang Z, Fitzgerald KA, Rothenfusser S, Endres S, Hartmann G, Hornung V. Selection of molecular structure and delivery of RNA oligonucleotides to activate TLR7 versus TLR8 and to induce high amounts of IL-12p70 in primary human monocytes. J Immunol. 2009 Jun 1;182(11):6824-6833.

PMID: 19454678

2. Heil F, Hemmi H, Hochrein H, Ampenberger F, Kirschning C, Akira S, Lipford G, Wagner H, Bauer S. Species-specific recognition of single-stranded RNA via toll-like receptor 7 and 8. Science. 2004 Mar 5;303(5663):1526-1529.

PMID: 14976262

3. Hornung V, Guenthner-Biller M, Bourquin C, Ablasser A, Schlee M, Uematsu S, Noronha A, Manoharan M, Akira S, de Fougerolles A, Endres S, Hartmann G. Sequence-specific potent induction of IFN-alpha by short interfering RNA in plasmacytoid dendritic cells through TLR7. Nat Med. 2005 Mar;11(3):263-270.

PMID: 15723075

4. Forsbach A, Nemorin JG, Völp K, Samulowitz U, Montino C, Müller C, Tluk S, Hamm S, Bauer S, Lipford GB, Vollmer J. Characterization of conserved viral leader RNA sequences that stimulate innate immunity through TLRs. Oligonucleotides. 2007 Winter;17(4):405-417.

PMID: 18072859

5. Furset G, Fløisand Y, Sioud M. Impaired expression of indoleamine 2, 3-dioxygenase in monocyte-derived dendritic cells in response to Toll-like receptor-7/8 ligands. Immunology. 2008 Feb;123(2):263-271.

PMID: 17725606

6. Gantier MP, Tong S, Behlke MA, Xu D, Phipps S, Foster PS, Williams BR. TLR7 is involved in sequence-specific sensing of single-stranded RNAs in human macrophages. J Immunol. 2008 Feb 15;180(4):2117-2124.

PMID: 18250417

7. Vollmer J, Tluk S, Schmitz C, Hamm S, Jurk M, Forsbach A, Akira S, Kelly KM, Reeves WH, Bauer S, Krieg AM. Immune stimulation mediated by autoantigen binding sites within small nuclear RNAs involves Toll-like receptors 7 and 8. J Exp Med. 2005 Dec 5;202(11):1575-1585.

PMID: 16330816

8. Sioud M. Single-stranded small interfering RNA are more immunostimulatory than their double-stranded counterparts: a central role for 2'-hydroxyl uridines in immune responses. Eur J Immunol. 2006 May;36(5):1222-1230.

PMID: 16609928

9. Forsbach A, Nemorin JG, Montino C, Müller C, Samulowitz U, Vicari AP, Jurk M, Mutwiri GK, Krieg AM, Lipford GB, Vollmer J. Identification of RNA sequence motifs stimulating sequence-specific TLR8-dependent immune responses. J Immunol. 2008 Mar 15;180(6):3729-3738.

PMID: 18322178

10. Diebold SS, Massacrier C, Akira S, Paturel C, Morel Y, Reis e Sousa C. Nucleic acid agonists for Toll-like receptor 7 are defined by the presence of uridine ribonucleotides. Eur J Immunol. 2006 Dec;36(12):3256-3267. PMID: 17111347

11. Negishi H, Endo N, Nakajima Y, Nishiyama T, Tabunoki Y, Nishio J, Koshiba R, Matsuda A, Matsuki K, Okamura T, Negishi-Koga T, Ichinohe T, Takemura S, Ishiwata H, Iemura SI, Natsume T, Abe T, Kiyonari H, Doi T, Hangai S, Yanai H, Fujio K, Yamamoto K, Taniguchi T. Identification of U11snRNA as an endogenous agonist of TLR7-mediated immune pathogenesis. Proc Natl Acad Sci U S A. 2019 Nov 19;116(47):23653-23661. doi: 10.1073/pnas.1915326116.

PMID: 31694883

12. Bourquin C, Schmidt L, Hornung V, Wurzenberger C, Anz D, Sandholzer N, Schreiber S, Voelkl A, Hartmann G, Endres S. Immunostimulatory RNA oligonucleotides trigger an antigen-specific cytotoxic T-cell and IgG2a response. Blood. 2007 Apr 1;109(7):2953-60.

PMID: 17132722

13. Bourquin C, Schmidt L, Lanz AL, Storch B, Wurzenberger C, Anz D, Sandholzer N, Mocikat R, Berger M, Poeck H, Hartmann G, Hornung V, Endres S. Immunostimulatory RNA oligonucleotides induce an effective antitumoral NK cell response through the TLR7. J Immunol. 2009 Nov 15;183(10):6078-86. doi: 10.4049/jimmunol.0901594.

PMID: 19890064

14. Forsbach A, Samulowitz U, Völp K, Hofmann HP, Noll B, Tluk S, Schmitz C, Wader T, Müller C, Podszuweit A, Lohner A, Curdt R, Uhlmann E, Vollmer J. Dual or triple activation of TLR7, TLR8, and/or TLR9 by single-stranded oligoribonucleotides. Nucleic Acid Ther. 2011 Dec;21(6):423-36. doi: 10.1089/nat.2011.0323.

PMID: 22196370

15. Green NM, Moody KS, Debatis M, Marshak-Rothstein A. Activation of autoreactive B cells by endogenous TLR7 and TLR3 RNA ligands. J Biol Chem. 2012 Nov 16;287(47):39789-99. doi: 10.1074/jbc.M112.383000. Epub 2012 Sep 27.

PMID: 23019335

16. Krüger A, Oldenburg M, Chebrolu C, Beisser D, Kolter J, Sigmund AM, Steinmann J, Schäfer S, Hochrein H, Rahmann S, Wagner H, Henneke P, Hornung V, Buer J, Kirschning CJ. Human TLR8 senses UR/URR motifs in bacterial and mitochondrial RNA. EMBO Rep. 2015 Dec;16(12):1656-63. doi: 10.15252/embr.201540861. Epub 2015 Nov 6.

PMID: 26545385

17. Sarvestani ST, Tate MD, Moffat JM, Jacobi AM, Behlke MA, Miller AR, Beckham SA, McCoy CE, Chen W, Mintern JD, O'Keeffe M, John M, Williams BR, Gantier MP. Inosine-mediated modulation of RNA sensing by Toll-like receptor 7 (TLR7) and TLR8. J Virol. 2014 Jan;88(2):799-810. doi: 10.1128/JVI.01571-13. Epub 2013 Nov 13.

PMID: 24227841
